# A Clinical Analysis and Classification Prediction of 145 Cases of Abdominal Wall Endometriosis

**DOI:** 10.1155/ogi/8033360

**Published:** 2026-08-24

**Authors:** Rong Gu, Li Huang, Hailiang Huang

**Affiliations:** ^1^ Department of Obstetrics and Gynecology, Anhui No.2 Provincial People’s Hospital, Hefei 230032, China; ^2^ School of Clinical Medicine, Anhui Institute of Medicine, Hefei 230032, China; ^3^ School of Clinical Medicine, Anhui Medical University, Hefei 230032, China, ahmu.edu.cn

**Keywords:** abdominal wall endometriosis, classification, endometriosis, subtype

## Abstract

**Objective:**

To retrospectively analyze the clinical data of 145 patients with abdominal wall endometriosis (AWE) secondary to cesarean section and to identify clinical indicators for predicting lesion classification.

**Methods:**

This was a retrospective observational study. We analyzed the general data and surgical details of 145 patients with AWE secondary to cesarean section, comparing three subtypes based on lesion invasion depth (Type I: subcutaneous fat; Type II: rectus muscle; Type III: peritoneal), two lesion patterns (solitary vs. multiple), and recurrence status.

**Results:**

Transverse incisions comprised 67.6% of cases, with higher implantation rates at the right end. Longitudinal incisions showed predominance at the lower end (66%). Type III patients were older and had longer operative times, greater blood loss, and larger lesions than Types I/II (all *p* < 0.05). Abdominal wall mass was the primary symptom across all types. Multiple lesion cases had a younger menarche age and longer operative time versus solitary lesions (*p* < 0.05). Recurrent cases had prolonged hospitalization (*p* < 0.05). MRI showed superior diagnostic concordance with surgery for lesion classification/sizing versus ultrasound (*p* < 0.05). ROC analysis identified menarche age ≤ 13.5 years as a significant predictor for multiple lesions.

**Conclusion:**

In post‐cesarean AWE, Type III lesions are associated with greater surgical complexity. MRI provides superior preoperative classification accuracy compared to ultrasound. An age at menarche ≤ 13.5 years is a significant predictor for multiple AWE lesions, offering a valuable tool for preoperative risk assessment.

## 1. Introduction

Endometriosis is a common gynecological disorder characterized by the presence of functional endometrial glands and stroma outside the uterine cavity, affecting approximately 10% of women of reproductive age [1]. Common implantation sites include pelvic organs and the peritoneum. Among extrapelvic endometriosis sites, abdominal wall endometriosis (AWE) is commonly described as the most frequent or one of the most frequent forms. It is defined as ectopic functional endometrial tissue located within the abdominal wall, usually superficial to or involving layers of the abdominal wall such as the subcutaneous tissue, fascia/rectus abdominis muscle, and sometimes the peritoneal layer. Clinically, AWE most often presents as a palpable abdominal wall mass and cyclic pain related to menstruation, especially in women with prior cesarean or other uterine surgery [2, 3]. AWE can be primary or secondary, with the latter often following gynecological or obstetric surgeries such as cesarean sections or hysterectomies. Cesarean scar endometriosis is a prevalent form [4]. The present study focuses exclusively on AWE secondary to cesarean section, which accounts for the majority of surgically treated AWE cases.

The reported incidence of AWE following cesarean section ranges from 0.03% to 0.45%, and appears to be increasing alongside rising cesarean section rates worldwide [5, 6]. In China, nationwide epidemiological data on AWE remain limited. However, AWE is increasingly recognized as an important postoperative complication associated with cesarean delivery, particularly in the context of persistently high cesarean section rates in China, which have generally been reported at about 39%–45% overall and can be higher in some regions or hospital settings [7, 8]. The main clinical manifestations are abdominal wall masses and cyclic pain related to menstruation, with rare cases of lesion rupture and bleeding [9]. Due to atypical symptoms and variable locations, AWE is often misdiagnosed as granulomas, cysts, lipomas, or malignancies, leading to confusion with surgical conditions [10].

Several risk factors have been associated with the development of AWE after cesarean section. Cesarean delivery itself is the major antecedent [2, 5], and AWE lesions are reported more often after transverse Pfannenstiel incisions, frequently at the scar corners, especially the right corner [11]; however, recent evidence does not clearly establish incision direction itself as an independent determinant of occurrence [2]. Higher maternal BMI, multiple cesarean deliveries, and obesity have been associated with AWE or its recurrence [5, 11, 12]. AWE usually appears after a latent interval of months to years following surgery [6, 13]. Early menarche, which prolongs the duration of endogenous estrogen exposure, has also been proposed as a potential risk factor, though its independent role in post‐cesarean AWE has not been firmly established [14, 15].

This study retrospectively analyzed the clinical data of 145 patients with AWE secondary to cesarean section to investigate their clinical presentations, subtype characteristics, and surgical management, and to explore clinical indicators for predicting lesion classification.

## 2. Materials and Methods

### 2.1. Data Source

We retrospectively reviewed the medical records of all patients diagnosed with AWE at the Department of Obstetrics and Gynecology, Anhui No. 2 Provincial People’s Hospital and the First Affiliated Hospital of Anhui Medical University between January 2014 and December 2024. A total of 174 patients with a diagnosis of AWE were initially identified.

Inclusion criteria were defined as follows: (1) history of cesarean section with pathologically confirmed AWE at the surgical scar site; (2) open resection of the lesion; and (3) complete medical records available for review.

Exclusion criteria were defined as follows: (1) incomplete medical records; (2) receipt of any hormonal therapy within 6 months prior to surgery, including leuprorelin, ethinylestradiol/cyproterone acetate (Diane‐35), and mifepristone; (3) concomitant endocrine or metabolic diseases; (4) pregnancy at the time of diagnosis; and (5) history of malignant tumors.

The following variables were collected from the medical records: patient age, menstrual history (age at menarche, menstrual cycle duration, menstrual period duration), initial symptoms (abdominal wall mass or pain), latent period (time from last cesarean section to first symptom onset), disease duration (time from symptom onset to surgical treatment), lesion characteristics (size, number, subtype), and surgical information (operative time, intraoperative blood loss, hospital stay).

To minimize selection bias, all consecutive patients meeting the inclusion criteria during the 10‐year study period were reviewed. To reduce information bias, a standardized data extraction form was used, and two authors independently cross‐checked the extracted data against the original medical records.

### 2.2. Ethics Statement

The study protocol was approved by the hospital’s Ethics Committee (Approval No. (R) 2025‐077). The requirement for individual patient informed consent was waived by the Ethics Committee due to the retrospective nature of the study and the use of anonymized clinical data.

### 2.3. Classification and Grouping

For the purpose of this retrospective study, all cases were reclassified according to the 2024 Chinese expert consensus on AWE [16]. This classification was based on a detailed review of the original surgical records and pathological reports, which contained sufficient information on the depth of lesion invasion to allow retrospective categorization. Lesions were classified by invasion depth as follows: (1) Type I (Subcutaneous fat type): lesion infiltrating to the rectus abdominis fascia; (2) Type II (Rectus muscle type): lesion invading the rectus abdominis muscle, not penetrating the peritoneum; and (3) Type III (Peritoneal type): lesion infiltrating to or through the peritoneum into the abdominal cavity. Based on lesion number, cases were categorized as Solitary or Multiple (≥ 2 lesions; classification based on the deepest involved site). The latent period was defined as the interval between the last surgery and the first symptom onset. Disease duration was the time from symptom onset to surgical treatment.

### 2.4. Surgical Method

All patients underwent surgical resection as part of their routine clinical care. The surgical approach was identified from the medical records. Preoperative localization was performed using color Doppler ultrasound or MRI. Under general or epidural anesthesia, abdominal wall ectopic lesion resection was performed with a resection margin of at least 1 cm beyond the lesion boundary, followed by thorough hemostasis. The wound was irrigated with 0.9% sodium chloride solution and closed with absorbable sutures. Throughout the 10‐year study period, open surgical resection remained the standard approach for AWE at both institutions. The use of preoperative MRI for lesion characterization increased over time, particularly in the later years of the study, while color Doppler ultrasound was consistently used throughout.

In complex cases involving deep peritoneal penetration or large abdominal wall defects, biological mesh was used for abdominal wall reconstruction at the surgeon’s discretion.

### 2.5. Statistical Analysis

Statistical analysis was performed using SPSS 26.0. Normally distributed continuous data are presented as mean ± standard deviation; nonnormally distributed data are presented as median (interquartile range). For comparisons between two groups, the independent samples *t*‐test was used for normal data and the Mann–Whitney *U* test for nonnormal data. For multi‐group comparisons, one‐way ANOVA with an LSD post hoc test was used for normal data, and the Kruskal–Wallis test with Bonferroni correction for nonnormal data. Categorical data are presented as frequency (percentage), and compared using the chi‐square test or Fisher’s exact test. ROC curve analysis was used to evaluate predictive performance. A *p* value < 0.05 was considered statistically significant.

## 3. Results

### 3.1. Analysis of General Data

**FIGURE 1:**
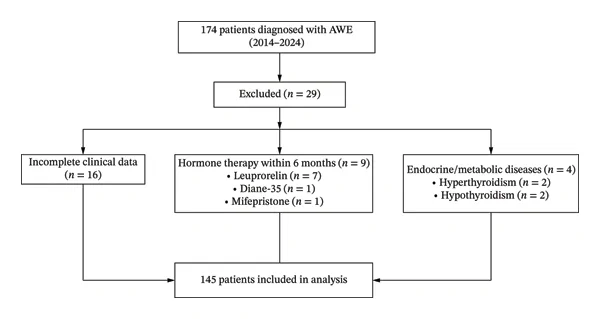
Flow diagram of patient selection.

A total of 174 patients with AWE were initially identified during the 10‐year study period. Of these, 16 were excluded due to incomplete medical records, 9 due to receipt of hormonal therapy within six months prior to surgery (leuprorelin, *n* = 7; Diane‐35, *n* = 1; mifepristone, *n* = 1), and 4 due to concomitant endocrine or metabolic diseases (hyperthyroidism, *n* = 2; hypothyroidism, *n* = 2). No patients were excluded for pregnancy or a history of malignant tumors. After applying all exclusion criteria, 29 patients were excluded, and 145 patients were included in the final analysis (Figure 1). All 145 included patients had complete data for the variables analyzed in this study. Among 145 AWE cases, transverse incisions accounted for 98 cases (67.6%). For transverse incisions, right‐sided lesions (44, 44.9%) were more common than left‐sided (40, 40.8%) and midline (14, 14.3%) lesions. There were 47 cases (32.4%) with longitudinal incisions, among which lower‐end lesions (31, 66.0%) were significantly more frequent than upper‐end (13, 27.7%) and midline (3, 6.4%) lesions. Patients with Type III AWE were significantly older than those with Types I and II (*p* < 0.05). See Table 1.

**TABLE 1:** Analysis of general data by lesion subtype.

Variable	Type I (*n* = 88)	Type II (*n* = 37)	Type III (*n* = 20)	*χ* ^2^/*F*/*H*	*p*
Age (years)	32.08 ± 4.36	31.38 ± 4.74	34.85 ± 4.91^e^ ^,^ ^f^	4.030^a^	0.020
Number of pregnancies	2.00 (1.00, 3.00)	2.00 (2.00, 3.00)	2.00 (2.00, 3.00)	1.824^b^	0.402
Number of cesarean sections	1.00 (1.00, 2.00)	1.00 (1.00, 2.00)	2.00 (1.00, 2.00)	3.521^b^	0.172
First vaginal delivery, *n* (%)	6 (6.8)	4 (10.8)	3 (15.0)	—^d^	0.407
Incision position, *n* (%)				—^d^	0.330
Transverse left	23 (26.1)	11 (29.7)	6 (30.0)		
Transverse right	27 (30.7)	14 (37.8)	3 (15.0)		
Transverse middle	12 (13.6)	1 (2.7)	1 (5.0)		
Longitudinal upper	6 (6.8)	3 (8.1)	4 (20.0)		
Longitudinal middle	3 (3.4)	0 (0)	0 (0)		
Longitudinal lower	17 (19.3)	8 (21.6)	6 (30.0)		
Weight (kg)	59.39 ± 9.41	56.05 ± 7.84	58.93 ± 9.26	1.808^a^	0.168
BMI (kg/m^2^)	22.96 ± 3.12	21.92 ± 2.88	22.82 ± 3.04	1.541^a^	0.218
Primary symptom, *n* (%)				0.070^c^	0.965
Mass	59 (67.0)	24 (64.9)	13 (65.0)		
Pain	29 (33.0)	13 (35.1)	7 (35.0)		

^a^One‐way ANOVA was used.

^b^The Kruskal–Wallis rank‐sum test was used.

^c^The chi‐square test was used.

^d^Fisher’s exact test was used.

^e^ *p* < 0.05 compared with Type I.

^f^ *p* < 0.05 compared with Type II.

### 3.2. Analysis of Lesion Subtype vs. Disease Course, Menstrual Cycle, and Surgery

No significant differences were found in menarche age, menstrual cycle duration, disease duration, or hospital stay among the different lesion subtypes (all *p* > 0.05). However, patients with Type III lesions had a significantly larger lesion size, longer operative time, and greater intraoperative blood loss compared to those with Type I and II lesions (all *p* < 0.05, Table 2). Among the 20 patients with Type III lesions, 3 required abdominal wall reconstruction with biological mesh. In one of these cases, the lesion was found to penetrate the peritoneum and communicate with the uterine cavity, presenting a particularly complex surgical scenario.

**TABLE 2:** Comparison of disease course, lesion size, and menstrual cycle by lesion subtype.

Variable	Type I (*n* = 88)	Type II (*n* = 37)	Type III (*n* = 20)	*H*	*p*
Age at menarche (years)	14.00 (13.00, 15.00)	14.00 (13.00, 14.00)	14.00 (13.00, 16.00)	0.332	0.847
Menstrual period (days)	7.00 (5.00, 7.00)	7.00 (5.00, 7.00)	7.00 (5.00, 7.00)	0.996	0.608
Menstrual cycle (days)	30.00 (28.00, 30.00)	30.00 (28.00, 30.00)	30.00 (28.00, 30.00)	2.964	0.227
Latent period (months)	36.00 (18.00, 60.00)	36.00 (17.00, 42.00)	36.00 (24.00, 66.00)	5.466	0.065
Course of disease (months)	22.00 (6.25, 36.00)	18.00 (6.00, 48.00)	12.00 (10.00, 24.00)	0.230	0.891
Lesion size (cm)	3.00 (2.00, 3.00)	3.00 (2.25, 4.00)	4.00 (3.50, 5.00)^a^ ^,^ ^b^	22.748	< 0.001
Operation time (min)	30.00 (25.00, 44.00)	35.00 (27.50, 54.00)	72.50 (52.00, 96.25)^a^ ^,^ ^b^	34.977	< 0.001
Intraoperative blood loss (mL)	5.00 (5.00, 10.00)	5.00 (2.00, 10.00)	20.00 (5.00, 40.00)^a^ ^,^ ^b^	15.115	0.001
Hospital stay (days)	5.00 (4.00, 6.00)	5.00 (4.00, 7.00)	6.00 (4.00, 8.00)	4.911	0.086

^a^ *p* < 0.05 compared with Type I.

^b^ *p* < 0.05 compared with Type II.

### 3.3. Analysis of Basic Data: Solitary vs. Multiple Lesions

In the solitary lesion group, Type I lesions accounted for the highest proportion (62.5%), whereas Type II was the most common subtype in the multiple lesion group (44.4%); however, the overall distribution of lesion subtypes did not differ significantly between the two groups (*p* = 0.173). Patients with multiple lesions had a significantly younger menarche age (*p* < 0.001) and longer operative time (*p* = 0.002) than those with solitary lesions. See Table 3.

**TABLE 3:** Analysis of basic data: solitary vs. multiple lesions.

Variable	Solitary lesions (*n* = 136)	Multiple lesions (*n* = 9)	*χ* ^2^/ *t*/*z*	*p*
Age (years)	32.24 ± 4.64	33.00 ± 4.74	−0.479^a^	0.633
Gravidity	2.00 (1.00, 3.00)	2.00 (2.00, 2.00)	−0.308^b^	0.758
Number of cesarean sections	1.00 (1.00, 2.00)	2.00 (1.00, 2.00)	−1.949^b^	0.051
Primary vaginal delivery, *n* (%)	12 (8.8)	1 (11.1)	< 0.001^c^	1.000
Menarche age (years)	14.00 (13.00, 15.00)	13.00 (12.00, 13.00)	−3.556^b^	< 0.001
Menstrual period (days)	7.00 (5.00, 7.00)	6.00 (5.00, 7.00)	−0.725^b^	0.469
Menstrual cycle (days)	30.00 (28.00, 30.00)	30.00 (29.00, 30.00)	−0.163^b^	0.871
Latent period (months)	36.00 (17.50, 48.00)	36.00 (24.00, 48.00)	−0.356^b^	0.722
Disease course (months)	19.00 (6.25, 36.00)	12.00 (7.00, 42.00)	−0.285^b^	0.776
Weight (kg)	58.36 ± 9.00	60.22 ± 10.39	−0.596^a^	0.552
BMI (kg/m^2^)	22.58 ± 3.02	24.13 ± 3.46	−1.481^a^	0.141
Lesion size (cm)	3.00 (2.00, 4.00)	3.50 (2.75, 4.00)	−1.531^b^	0.126
Operation time (min)	35.00 (26.25, 50.00)	80.00 (38.50, 87.50)	−3.081^b^	0.002
Intraoperative blood loss (mL)	5.00 (5.00, 10.00)	10.00 (5.00, 20.00)	−1.386^b^	0.166
Hospital stay (days)	5.00 (4.00, 6.00)	5.00 (5.00, 7.50)	−1.167^b^	0.243
Lesion subtype, *n* (%)			—^d^	0.173
Type I	85 (62.5)	3 (33.3)		
Type II	33 (24.3)	4 (44.4)		
Type III	18 (13.2)	2 (22.2)		

^a^The independent samples *t*‐test was used.

^b^The Mann–Whitney *U* test was used.

^c^The chi‐square test (with continuity correction) was applied.

^d^Fisher’s exact test was used.

### 3.4. Predictive Value of Menarche Age for Multiple Lesions

**FIGURE 2:**
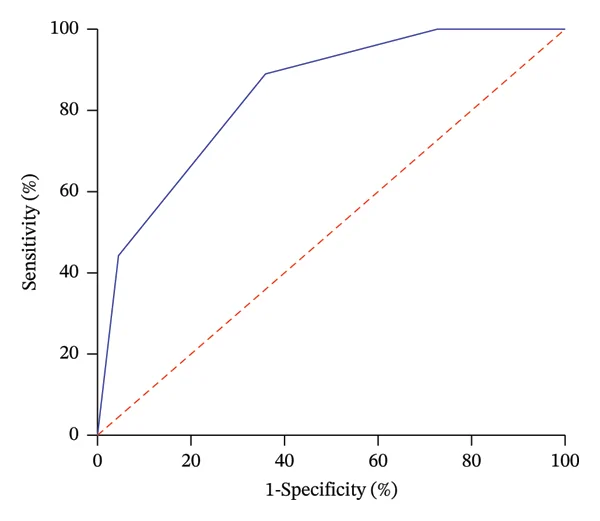
ROC curve of menarche age for predicting multiple lesions.

Using disease status (multiple = 1, solitary = 0) as the state variable, ROC curve analysis showed that menarche age had significant discriminatory value for predicting multiple lesions (AUC = 0.840, 95% CI: 0.719–0.960, *p* = 0.001). At a cutoff value of ≤ 13.5 years, specificity was 0.640, sensitivity was 0.889, and the maximum Youden index was 0.529. See Table 4 and Figure 2.

**TABLE 4:** Predictive performance of menarche age for multiple lesions.

Variable	Cut‐off value	AUC	95% CI		*p*	Specificity	Sensitivity	Youden’s index
			Lower limit	Upper limit				
Menarche age	13.5	0.840	0.719	0.960	0.001	0.640	0.889	0.529

### 3.5. Clinical Data Analysis: Recurrent vs. Non–recurrent Patients

Recurrent patients had a significantly longer hospital stay than non–recurrent patients (*p* < 0.05). No other significant differences were observed between the groups (*p* > 0.05). See Table 5.

**TABLE 5:** Analysis of basic data: recurrent vs. nonrecurrent patients.

Variable	Nonrecurrent (*n* = 137)	Recurrent (*n* = 8)	*χ* ^2^/ *t*/*z*	*p*
Age (years)	32.19 ± 4.61	33.88 ± 5.00	−1.001^a^	0.319
Gravidity	2.00 (1.00, 3.00)	2.00 (1.25, 4.00)	−0.551^b^	0.582
Number of cesarean sections	1.00 (1.00, 2.00)	1.50 (1.00, 2.00)	−0.832^b^	0.405
Primary vaginal delivery, *n* (%)	13 (9.5)	0 (0)	0.076^c^	0.782
Menarche age (years)	14.00 (13.00, 15.00)	14.00 (12.25, 14.00)	−0.669^b^	0.504
Menstrual period (days)	7.00 (5.00, 7.00)	7.00 (5.25, 7.00)	−0.404^b^	0.687
Menstrual cycle (days)	30.00 (28.00, 30.00)	30.00 (28.50, 30.00)	−0.565^b^	0.572
Latent period (months)	36.00 (19.00, 48.00)	30.00 (24.00, 66.00)	−0.249^b^	0.803
Disease course (months)	12.00 (6.00, 36.00)	36.00 (9.00, 79.50)	−1.129^b^	0.259
Weight (kg)	58.42 ± 9.21	59.44 ± 6.38	−0.309^a^	0.758
BMI (kg/m^2^)	22.64 ± 3.12	23.25 ± 1.80	−0.540^a^	0.590
Lesion size (cm)	3.00 (2.00, 4.00)	3.50 (2.63, 4.00)	−1.067^b^	0.286
Operation time (min)	35.00 (29.00, 53.50)	28.00 (25.00, 91.75)	−0.517^b^	0.605
Intraoperative blood loss (mL)	5.00 (5.00, 10.00)	5.00 (5.00, 5.00)	−0.739^b^	0.460
Hospital stay (days)	5.00 (4.00, 6.00)	7.00 (5.50, 10.50)	−2.439^b^	0.015
Lesion subtype, *n* (%)			—^d^	0.510
Type I	84 (61.3)	4 (50.0)		
Type II	35 (25.5)	2 (25.0)		
Type III	18 (13.1)	2 (25.0)		

^a^The independent samples *t*‐test was used.

^b^The Mann–Whitney *U* test was used.

^c^The corrected chi‐square test was used.

^d^Fisher’s exact test was used.

### 3.6. Comparison of Surgical Exploration, MRI, and Color Doppler Ultrasound Results

In 42 patients who underwent all three assessments (ultrasound, MRI, surgery), the Friedman test showed that MRI had significantly higher concordance with surgical findings for lesion classification compared to ultrasound (*p* < 0.05). For lesion diameter measurement, MRI values were closer to surgical measurements than ultrasound values (*p* < 0.05). See Table 6.

**TABLE 6:** Analysis of lesion classification and size by surgical group, MRI group, and ultrasound group.

Variable	Surgical group (*n* = 42)	MRI group (*n* = 42)	Ultrasound group (*n* = 42)	*χ* ^2^	*p*
Lesion subtype, *n* (%)				36.286	< 0.001
Type I	6 (14.3)	6 (14.3)	22 (52.4)^a^ ^,^ ^b^		
Type II	26 (61.9)	27 (64.3)	17 (40.5)^a^ ^,^ ^b^		
Type III	10 (23.8)	9 (21.4)	3 (7.1)^a^ ^,^ ^b^		
Lesion size (cm)	4.00 (3.00, 4.50)	3.95 (3.00, 4.55)	2.85 (2.15, 3.90)^a^ ^,^ ^b^	34.087	< 0.001

^a^ *p* < 0.05 compared with the surgical group.

^b^ *p* < 0.05 compared with the MRI group.

## 4. Discussion

Our understanding of AWE can be traced back to the first case reported by Meyer in 1903. Cesarean section has long been considered a primary predisposing factor, with the widely accepted mechanism being the translocation and implantation of endometrial tissue into the abdominal wall during surgery. However, this theory has limitations. For instance, why do many women who undergo cesarean sections not develop AWE? How does primary AWE without a surgical history occur? Consequently, subsequent theories have emerged, including coelomic epithelial metaplasia, dissemination of endometrial fragments via blood or lymphatic vessels, the influence of local inflammatory responses, and even genetic factors [7]. This study retrospectively analyzed the clinical data of 145 patients with AWE secondary to cesarean section to investigate their clinical presentations, subtype characteristics, and surgical management and to explore clinical indicators for predicting lesion classification.

Transverse (Pfannenstiel incision) and longitudinal incisions are the two main types of cesarean section incisions. We found that the incidence of AWE was higher with transverse incisions than longitudinal ones, and it occurred more frequently at the right edge of the Pfannenstiel incision and the lower end of longitudinal incisions. We speculate that this disparity may be related to the following mechanisms. First, transverse incisions transect more blood vessels, creating a more favorable environment for angiogenesis. Angiogenesis is a key factor in the development of endometriosis [17]. As abdominal wall vessels are predominantly longitudinally oriented, a transverse incision severs more vessels. During the repair process, a rich vascular network forms, providing a favorable environment for any residual endometrial fragments. More importantly, activated platelets can trigger the TGF‐β1/Smad3 signaling pathway via cytokine responses [18], promoting tissue fibrosis and establishing a foundation for the establishment and maintenance of ectopic foci. Second, surgical manipulation may lead to the “deposition” of endometrial fragments. During delivery, the common practice of delivering the fetal head from the mother’s right side, combined with the effect of gravity on irrigation fluid, can easily create a “sump” area in the incision region. Consequently, the ends of transverse incisions (especially the right end) and the lower end of longitudinal incisions are more prone to accumulate endometrial fragments, increasing the long‐term risk of AWE. Moreover, although our study did not find a significant difference in BMI across the three lesion subtypes, or between solitary and multiple lesion groups, the potential exacerbating role of obesity should not be overlooked. Published evidence indicates that obesity, defined as BMI ≥ 25 kg/m^2^, is an independent risk factor for AWE [19]. A thickened abdominal wall fat layer can increase operative complexity, may limit effective clearance of endometrial debris from the surgical field, and is associated with impaired wound healing [20, 21]. The relatively homogeneous BMI range in our cohort (mean 22–24 kg/m^2^) and the limited sample size may have precluded the detection of significant differences. These two aspects—impaired irrigation and delayed wound healing—synergistically create conditions conducive to the implantation and growth of ectopic endometrium.

An abdominal wall mass is often the primary complaint leading patients to seek medical attention. In our study of 145 patients, this proportion was nearly two‐thirds, consistent with the findings of Zhang et al. [22]. Regarding imaging, economical and convenient color Doppler ultrasound is often chosen for initial screening. Our comparative analysis of data from 42 patients who underwent color Doppler ultrasound, MRI, and surgical exploration showed that MRI findings regarding lesion diameter and infiltration depth were closer to surgical findings than ultrasound, highlighting the anatomical advantages of MRI [23].

Radical lesion resection remains the current gold standard, requiring a resection margin of at least 1 cm beyond the lesion. In the comparative analysis of recurrent versus nonrecurrent patients, the recurrence group had a significantly longer hospital stay [24, 25]. While the causes of AWE recurrence are likely multifactorial, a longer hospital stay may reflect more extensive surgical dissection or more complex primary surgeries. However, given the small number of recurrent cases in our cohort (*n* = 8) and the retrospective nature of this study, no definitive conclusions about the causation of recurrence can be drawn. Surgical difficulty escalates with the lesion subtype, progressing from the relatively superficial Types I and II to Type III AWE involving the peritoneum. Operative time was significantly longer, intraoperative blood loss was significantly greater, and surgical complexity increased accordingly. Types II and III may even require biological mesh for abdominal wall reconstruction to prevent incisional hernias. As noted in the Results, three Type III patients required abdominal wall reconstruction with biological mesh, including one case in which the lesion penetrated the peritoneum and communicated with the uterine cavity, illustrating the surgical challenges posed by deep infiltrating AWE. While open surgery remains the standard approach used in our cohort, minimally invasive techniques—such as single‐port laparoscopy and robot‐assisted systems—have been reported in the literature as valuable options for challenging cases, including lesions distant from the original incision, thick abdominal wall fat, deep infiltration, and multiple lesions [26]. However, these techniques were not evaluated in our study.

As one of many estrogen‐dependent diseases, endometriosis involves ectopic endometrial tissue that often exhibits higher estrogen receptor levels compared to normal endometrium. In this study, the age of onset for Type III AWE was higher than for the other two types, supporting the notion that longer exposure to estrogen enhances the invasiveness of ectopic endometrium and increases the long‐term risk of malignant transformation [27]. No significant differences were observed in menstrual characteristics or menarche age among the three AWE subtypes. Solitary and multiple lesions were classified primarily based on surgical exploration. Solitary lesions were predominantly Type I (62.5%), while among the 9 multiple lesion cases, Type II was most common (44.4%). Surgery in the multiple lesion group took significantly longer. Furthermore, menarche age was significantly younger in the multiple lesion group compared to the solitary lesion group. Menarche in females marks the onset of high‐level endogenous estrogen release. Early menarche, late menopause, and obesity increase the duration of endogenous estrogen exposure, also elevating the risk of developing endometriosis and cancers (e.g., breast cancer, endometrial cancer) [28–30]. Among these, early menarche was identified in our study as a significant predictor for multiple AWE lesions, with menarche age ≤ 13.5 years showing good discriminatory ability. However, whether it constitutes an independent risk factor requires confirmation through multivariable analyses in larger studies.

This study has several limitations. First, its retrospective design and relatively small sample size, particularly in the multiple lesion and recurrent subgroups, limit the statistical power to detect differences and the generalizability of the findings. Second, the study was conducted at only two hospitals in a single province of China, which may limit the applicability of the findings to other populations or healthcare settings. Third, given the 10‐year study period (2014–2024), there were inevitable changes in diagnostic imaging practices; in particular, the use of preoperative MRI for lesion characterization increased over time, which may have influenced the accuracy of preoperative lesion classification in the later years of the study. Although open surgical resection remained the standard approach throughout the study period, subtle refinements in surgical technique and perioperative care over the decade may also have affected operative time, intraoperative blood loss, and length of hospital stay. Additionally, some potentially relevant variables—such as detailed surgical history, timing of uterine closure during cesarean section, and postoperative wound complications—were not consistently available in the medical records and could not be analyzed. Finally, as all patients in this study had AWE secondary to cesarean section, the findings may not be directly applicable to primary AWE or AWE secondary to other surgical procedures.

In conclusion, in this cohort of patients with AWE secondary to cesarean section, Type III lesions were associated with greater surgical complexity, including longer operative time and greater intraoperative blood loss. Preoperative MRI demonstrated superior accuracy over ultrasound for evaluating lesion depth and size. Furthermore, an age at menarche of ≤ 13.5 years was identified as a significant predictor for the presence of multiple AWE lesions, suggesting that early menarche may serve as a useful marker for identifying patients at higher risk. For patients diagnosed with AWE, preoperative assessment incorporating menarche age and MRI findings may assist in anticipating lesion subtype and multiplicity, which could inform the choice of surgical approach, the potential need for abdominal wall reconstruction, and multidisciplinary surgical planning. Future prospective studies with larger sample sizes are warranted to validate these findings.

## Author Contributions

Conceptualization, methodology, and data curation: Li Huang; validation and formal analysis: Hailiang Huang; writing–original draft preparation and writing–review and editing: Rong Gu.

## Funding

This work was supported by the Provincial‐Level Quality Engineering Project for Higher Education Institutions of Anhui Province under Grant No. 2024cxtd354 and the Key Scientific Research Project of the Department of Education of Anhui Province (Natural Science) under Grant No. 2025AHGXZK31507.

## Disclosure

All authors have read and agreed to the published version of the manuscript.

## Ethics Statement

The study was conducted in accordance with the Declaration of Helsinki and approved by the Ethics Committee of Anhui No. 2 Provincial People’s Hospital (Approval No. (R) 2025‐077).

## Consent

The requirement for individual patient informed consent was waived by the Ethics Committee due to the retrospective nature of the study and the use of anonymized clinical data.

## Conflicts of Interest

The authors declare no conflicts of interest.

## Data Availability

The data that support the findings of this study are available from the corresponding author upon reasonable request.
